# *Pm*VRP15, a Novel Viral Responsive Protein from the Black Tiger Shrimp, *Penaeus monodon*, Promoted White Spot Syndrome Virus Replication

**DOI:** 10.1371/journal.pone.0091930

**Published:** 2014-03-17

**Authors:** Tipachai Vatanavicharn, Adisak Prapavorarat, Phattarunda Jaree, Kunlaya Somboonwiwat, Anchalee Tassanakajon

**Affiliations:** 1 Applied Analytical Chemistry Research Unit, Department of Chemistry, Faculty of Science, King Mongkut's Institute of Technology Ladkrabang, Bangkok, Thailand; 2 Center of Excellence for Molecular Biology and Genomics of Shrimp, Department of Biochemistry, Faculty of Science, Chulalongkorn University, Bangkok, Thailand; Uppsala University, Sweden

## Abstract

Suppression subtractive hybridization of *Penaeus monodon* hemocytes challenged with white spot syndrome virus (WSSV) has identified the viral responsive gene, *Pm*VRP15, as the highest up-regulated gene ever reported in shrimps. Expression analysis by quantitative real time RT-PCR revealed 9410–fold up-regulated level at 48 h post WSSV injection. Tissue distribution analysis showed that *Pm*VRP15 transcript was mainly expressed in the hemocytes of shrimp. The full-length cDNA of *Pm*VRP15 transcript was obtained and showed no significant similarity to any known gene in the GenBank database. The predicted open reading frame of *Pm*VRP15 encodes for a deduced 137 amino acid protein containing a putative transmembrane helix. Immunofluorescent localization of the *Pm*VRP15 protein revealed it accumulated around the nuclear membrane in all three types of shrimp hemocytes and that the protein was highly up-regulated in WSSV-infected shrimps. Double-stranded RNA interference-mediated gene silencing of *Pm*VRP15 in *P. monodon* significantly decreased WSSV propagation compared to the control shrimps (injected with GFP dsRNA). The significant decrease in cumulative mortality rate of WSSV-infected shrimp following *Pm*VRP15 knockdown was observed. These results suggest that *Pm*VRP15 is likely to be a nuclear membrane protein and that it acts as a part of WSSV propagation pathway.

## Introduction

White spot syndrome, caused by the white spot syndrome virus (WSSV), is the most serious viral disease in penaeid shrimps causing 100% mortality post infection [1]. In the last two decades outbreaks of this virus in commercial shrimp aquaculture farms have been reported in Asia and America [1]–[7]. WSSV has bacilliform, enveloped, non-occluded virions containing a double stranded (ds)DNA genome [8]–[10]. The virus has a wide host range, where more than 93 species of arthropods have been reported as hosts or carriers of WSSV [11]. The mechanism of WSSV infection and propagation in the host cell remains unknown in spite of its severe impact on the shrimp farming and that understanding of the virus-host interaction is likely to be the key point in developing strategies to prevention of this disease outbreak.

The invasion of WSSV into the penaeid shrimps affects their immune defense responses. The molecular changes associated at the gene transcript and protein expression levels in the shrimp immune system have been investigated using expressed sequenced tag (EST) [12]–[13], DNA microarray [14]–[18] and proteomic [19]–[20] analyses. The, up-regulated gene transcripts or proteins have been further characterized for their potential role in both the cellular and humoral immunity (defense responses) of shrimps in response to WSSV infection. These were found to include the antimicrobial peptides, prophenol oxidase (proPO) system, oxidative stress, proteinases and proteinase inhibitors [21]. Moreover, three of the major immune responses (phagocytosis, apoptosis and the proPO cascade) have been compared to study their role in the antiviral defense system [22].

The novel proteins that are up-regulated in shrimps following WSSV infection are typically viewed as interesting molecules to characterize their function in the shrimp immune system. For example, the novel viral responsive protein, hemocyte homeostasis-associated protein (HHAP), was found to be highly up-regulated at both the transcript and protein levels in WSSV-infected shrimp hemocytes. Silencing of this gene in *Penaeus monodon* (*Pm*HHAP) by dsRNA-interference (RNAi) caused damage to shrimp hemocytes and a severe decrease in their numbers, suggesting the important role of *Pm*HHAP in hemocyte homeostasis [23]. Suppression subtractive hybridization (SSH) and microarray analyses (our unpublished data) of WSSV-challenged *P. monodon* hemocytes identified the novel viral responsive protein (VRP) *Pm*VRP15 as one of the most highly up-regulated genes in the acute phase of WSSV-infected hemocytes. Herein, we attempt to characterize the function of *Pm*VRP15 from *P. monodon* by RNAi-mediated gene silencing. Fluorescence-labeling along with confocal laser scanning microscopy (CLSM) was used to examine the localization of *Pm*VRP15 in shrimp hemocytes. Overall, the likely importance of this novel protein in promoting viral propagation was suggested.

## Materials and Methods

### Animal cultivation

Specific pathogen free black tiger shrimps, *P. monodon*, of about 20- and 3-g body weight, were obtained from a commercial shrimp farm in Nakhon Si Thammarat Province, Thailand. The animals were reared in laboratory tanks at ambient temperature (28±4°C), and maintained in aerated water with a salinity of 20 ppt for at least 7 d before use.

### Identification of a full-length cDNA of *Pm*VRP15 using 5′ Rapid Amplification of cDNA End (5′ RACE)

The partial sequence of the *Pm*VRP15 cDNA was initially obtained from the SSH library of WSSV-challenged *P. monodon* hemocytes, and then extended by 5′ RACE. The hemolymph of ∼20 g body weight shrimps was drawn from the ventral sinus using a sterile 1-mL syringe with 150 μL of 10% (w/v) sodium citrate solution. The hemolymph was immediately centrifuged at 5000×g for 5 minutes at 4 °C to separate the hemocytes (pellet) from the plasma (supernatant). Total RNA was isolated from the hemocytes using the TRI Reagent (Molecular Research Center) according to the manufacturer's protocol. A full-length cDNA of *Pm*VRP15 was determined using The SMART RACE cDNA Amplification Kit (Clontech) and the GSP-RACE primer (Table 1), according to the manufacturer's instruction. The RACE product was purified using a NucleoSpin Extract II kit (Clontech) according to manufacturer's protocol, and cloned into the RBC T&A Cloning Vector (RBC Bioscience). Then, the recombinant plasmid was transformed into *Escherichia coli* DH5α competent cells (RBC Bioscience). The positive clones were commercially sequenced by Macrogen INC., South Korea. The nucleotide sequences of SSH clone and RACE fragment were then assembled and searched against the NCBI database.

**Table 1:** Nucleotide sequences of the PCR primers used in this study.

Primer name	Sequence (5′→3′)
GSP-RACE	CGCCGCTCGCAGCTTCTTCTCTTGACAC
*Pm*VRP15F	CGATCACCACTCTCGTTCTT
*Pm*VRP15R	GTACTAACAGCGAACCCATC
*Pm*VRP15-RTF	CGTCCTTCAGTGCGCTTCCATA
*Pm*VRP15-RTR	ACAGCGACTCCAAGGTCTACGA
EF-1-F	GGTGCTGGACAAGCTGAAGGC
EF-1-R	CGTTCCGGTGATCATGTTCTTGATG
β-actin-F	GAACCTCTCGTTGCCGATGGTG
β-actin-R	GAAGCTGTGCTACGTGGCTCTG
r*Pm*VRP15-*Nco*IF	ATCGCCATGGGCATGTTAACAGAGGACTTA
r*Pm*VRP15-*Xho*IR	ATCGCTCGAGATGCTCTACTGACATGTTGTG
GFP-F	ATGGTGAGCAAGGGGGAGGA
GFP-R	TTACTTGTACAGCTCGTCCA
GFP-FT7	GGATCCTAATACGACTCACTATAGGATGGTGAGCAAGGGGGAGGA
GFP-RT7	GGATCCTAATACGACTCACTATAGG TTACTTGTACAGCTCGTCCA
*Pm*VRP15- T7-F	GGATCCTAATACGACTCACTATAGGCGCGACCGAGCCAAGAG
*Pm*VRP15- T7-R	GGATCCTAATACGACTCACTATAGGTGAGCTGACGGAAGGCC
*Pm*VRP15-F	TCACTCTTTCGGTCGTGTCG
*Pm*VRP15-R	CCACACACAAAGGTGCCAAC
VP28-qrt-F	GGGAACATTCAAGGTGTGGA
VP28-qrt-R	GGTGAAGGAGGAGGTGTTGG
ie1-qrt-F	AGCAAGTGGAGGTGCTATGT
ie1-qrt-R	CCATGTCGATCAGTCTCTTC
477-qrt-F	GGCCAAGTCATGGAGATCTA
477-qrt-R	CCATCCACTTGGTTGCAGTA

### Analysis of *Pm*VRP15 transcript expression in shrimp tissues

*Pm*VRP15 transcript expression levels in different tissues were qualitatively assayed by reverse transcriptase-PCR (RT-PCR). The different tissues of ∼20 g body weight shrimps such as antennal gland, epipodite, eye stalk, gill, heart, hemocytes, hepatopancreas, intestine and lymphoid, were collected from uninfected shrimps, and then total RNA was extracted from each tissue using the TRI Reagent (Molecular Research Center). After DNase I (Fermentas) treatment, the total RNA (1 μg) was first converted to single-stranded (ss)cDNA with the ImPromp-II reverse transcription system (Promega) according to the manufacturer's instruction. Then, in the second stage, *Pm*VRP15 transcript levels in each tissue were identified by PCR using 1 μL of the cDNA as a template with the *Pm*VRP15F/R primers (Table 1). The EF-1α gene fragment was amplified using the EF-1-F/R primers (Table 1) as an internal control. The PCR thermal cycling conditions consisted of 94°C for 3 min, followed by 35 (for *Pm*VRP15) or 27 (for EF-1α) cycles of 95°C for 30 s, 58°C for 30 s and 72°C for 30 s, and then a final extension at 72°C for 5 min. The PCR product was resolved by 1.5% (w/v) agarose-TBE gel electrophoresis and visualized by uv-transillumination following staining with ethidium bromide.

### *Pm*VRP15 mRNA expression in unchallenged- and WSSV-challenged shrimp hemocytes

WSSV was prepared from the gills of WSSV-challenged *P. monodon* as previously described [24], and then diluted in lobster hemolymph medium (LHM). Then 100 μL of the diluted WSSV suspension (∼80 viral copies/μL) was injected into each shrimp (∼20 g body weight), a viral dose that had been previously determined as that which would induce a cumulative mortality of ∼50% within 3 d post-injection. Control shrimps were likewise injected but with 100 μL of virus-free LHM. Hemocytes of shrimps (three individuals each) were collected at 24, 48 and 72 h post-infection (hpi) as above. *Pm*VRP15 transcript levels were then assayed by quantitative real time RT-PCR (qRT-PCR) as follows. Total RNA was then extracted from the hemocytes and used to synthesize sscDNA as above, whilst the qRT-PCR was performed with an equal amount of cDNAs in an iCycler iQ Real-Time Detection system using an IQ SYBR Green Supermix (Bio-Rad) and the *Pm*VRP15-RTF/R and β-actin-F/R primers (Table 1). Thermal cycling was performed as 95°C for 9 min, 40 cycles of 95°C for 30 s, 60°C for 30 s and 72°C for 45 s. The results are presented as the average relative expression ratio of *Pm*VRP15 transcript levels in the hemocytes of the sample (WSSV-challenged) shrimp versus the control (unchallenged) shrimp, after normalization to the transcript levels of the reference gene, β-actin. These relative expression ratios of *Pm*VRP15 gene were calculated as previously described [25].

### Production of recombinant (r)PmVRP15 (as a r(His)_6_-PmVRP15 chimera) in *E. coli*

The cDNA encoding for *Pm*VRP15 was PCR amplified from the *P. monodon* hemocyte cDNA as above using the gene specific primers *Pm*VRP15-*Nco*I/*Xho*I primers (Table 1) that contain 5′ flanking sequences with a *Nco*I and *Xho*I restriction site, respectively. The PCR product was double digested with *Nco*I and *Xho*I (New England Biolabs) and cloned in frame into the likewise double digested pET22-b. The ligation mixture was transformed into *E. coli* stain XL-1-blue. A single ampicillin resistant clone was selected, cultured and the recombinant plasmid was extracted and retransformed into the expression host, *E. coli* stain BL21(DE3). The recombinant plasmid was sequenced to confirm the correctness of the sequences. Then a selected recombinant clone in the expression host was cultured and induced with 1 mM IPTG for 4 h to over-produce the r(His)_6_-*Pm*VRP15. The cell pellet was collected by centrifugation at 8000×g for 10 min, resuspended in phosphate buffered saline (PBS pH 7.4) and sonicated with a Bransonic 32 (Bandelin) for 4 min. Inclusion bodies were collected by centrifugation at 10,000 rpm for 20 min to remove the supernatant. The inclusion body pellet was then dissolved in 8 M urea in PBS. The r(His)_6_-*Pm*VRP15 protein was purified using a Nickel-NTA column (GE healthcare), as per the manufacturer's protocol, and the resulting eluate dialyzed against distilled water. The protein was analyzed using SDS-PAGE, with the protein concentration determined using the Bradford method [26].

### Rabbit serum and anti-*Pm*VRP15 immune serum

Rabbit polyclonal antiserum against the purified r(His)_6_-*Pm*VRP15 protein (2 mg) was prepared commercially by the Biomedical Technology Research Unit, Faculty of Associated Medical Sciences, Chiang Mai University, Thailand.

### Western-blot analysis of *Pm*VRP15 protein in control and WSSV-infected *P. monodon* hemocytes

Hemocytes were collected from 48 hpi saline- or WSSV- injected shrimps as above. The hemocytes were homogenated in PBS and centrifuged to collect the supernatant. The protein concentration of the hemocyte lysate (HLS) was measured by the Bradford method [26]. Seventy μg of HLS protein (per lane) was subjected to SDS-PAGE (12% (w/v) acrylamide resolving gel) resolution, transferred to nitrocellulose membrane and then the *Pm*VRP15 and β-actin protein was detected by Western-blot analysis using purified rabbit polyclonal anti-r*Pm*VRP15 and mouse anti-actin (Millipore) antibodies. The positive band was detected by secondary antibodies conjugated with horseradish peroxidase (brown color) for mouse antibody or alkaline phosphatase (purple color) for rabbit antibody.

### Immunolocalization of *Pm*VRP15 protein in *P. monodon* hemocytes

The hemolymph was collected from control and WSSV-injected shrimps at 6, 24 and 48 hpi, as well as from moribund shrimps, and immediately fixed by incubation in 4% (w/v) paraformaldehyde at room temperature for 10 min. The fixed hemocytes were washed in PBS (centrifugation stage at 800×*g* at 4°C for 10 min) and resuspended in PBS. About 10^6^ hemocytes were attached onto each SuperFrost microscope slide by centrifugation at 1000×*g* for 10 min. Slides were blocked in 10% (v/v) fetal bovine serum in PBS at room temperature for 1 h and then probed with purified rabbit polyclonal antibody specific to *Pm*VRP15 and purified mouse monoclonal antibody specific to VP28 (WSSV capsid protein) for 1 h at room temperature and washed three times in 0.05% (v/v) Tween-20 in PBS to remove non-specific binding. The Alexa 488-conjugated goat anti-rabbit IgG and Alexa 568-conjugated goat anti-mouse IgG secondary antibodies (Invitrogen) were applied to the slides and incubated at room temperature for 1 h. The slides were then washed three times as above, incubated with TO-PRO-3 iodide (Molecular Probes) to stain the nuclear DNA and then washed once with PBS. Mounting medium, ProLong Gold antifade (Molecular Probes), was applied and the slides were examined by CLSM (Olympus). Bright field and fluorescence images were collected for the analyses.

### Production of *Pm*VRP15 and GFP dsRNA

The dsRNA specific to the *Pm*VRP15 gene transcript was prepared using the *Pm*VRP15-recombinant plasmid as a template for producing the sense and anti-sense DNA templates by *in vitro* transcription. DNA templates containing the T7 promoter sequence at the 5′-end were generated by PCR using the oligonucleotide primers *Pm*VRP15-T7-F and *Pm*VRP15-R (Table 1) for the sense strand template, and *Pm*VRP15-F and *Pm*VRP15-T7-R (Table 1) for the antisense strand template. In addition, dsRNA of the green fluorescent protein (GFP), the negative control, was prepared from the pEGFP-1 vector (Clontech) as the PCR template using the GFP-FT7 and GFP-R primers (Table 1) for the sense strand template, and the GFP-F and GFP-RT7 primers (Table 1) for the antisense strand template. The PCR was performed at 94°C for 3 min, followed by 30 cycles of 94°C for 30 s, 58°C for 30 s and 72°C for 30 s, and then a final extension at 72°C for 5 min. Each template was *in vitro* transcribed using the T7 RiboMAX Express RNAi System (Promega), according to the manufacturer's instruction, to produce the two complementary ssRNAs. Then, equal amounts of each of the complementary ssRNAs were mixed together and incubated at 70°C for 10 min, and slowly cooled down at room temperature to allow annealing to form dsRNA. The respective *Pm*VRP15 or GFP dsRNA solution was treated with 2 units (U) of RQ1 RNase-free DNase (Promega) at 37°C for 30 min, and then purified by standard phenol-chloroform extraction.

### *Pm*VRP15 gene knockdown in hemocyte of WSSV-infected shrimp

*P. monodon* shrimps of approximately 3 g body weight were divided into two groups of three individuals each. The first (control) group was injected with 10 μg/g shrimp of GFP-dsRNA, whilst the second group (*Pm*VRP15 knockdown) was injected with 10 μg/g shrimp *Pm*VRP15-dsRNA. After 24 h, 10 μg/g shrimp *Pm*VRP15-dsRNA or dsGFP was mixed with 30 μL of the 10,000-fold diluted WSSV solution (a dose that causes 100% mortality of shrimps in 3 dpi) and injected into the respective groups of shrimps. Hemocytes of individual shrimps were collected at 24 hpi, and total RNA was extracted as above and treated with 1 U of RQ1 RNase-free DNase (Promega) to remove any residual DNA contamination. An equal amount of DNA-free total RNA from three shrimps was pooled and from this 1 μg of total RNA was used for the first stage RT-PCR cDNA synthesis using the RevertAid First Strand cDNA Synthesis kit (Thermo Scientific).

To confirm the *Pm*VRP15 gene transcript knockdown, RT-PCR was performed. The *Pm*VRP15-F/R primers (Table 1) were used (100 nM) along with the EF-1α gene as an internal control using the EF-1-F/R primer pair (Table 1). The PCR conditions were 94°C for 1 min, followed by 27 cycles of 95°C for 30 s, 58°C for 30 s, and 72°C for 30 s, and then a final extension at 72°C for 5 min. The PCR products were analyzed by 1.5% (w/v) agarose gel electrophoresis.

The hemocyte of WSSV-infected *Pm*VRP15 gene knockdown shrimp and of the control was collected from 5 individuals. After protein extraction, protein lysate from each individual were pooled. *Pm*VRP15 protein expression after *Pm*VRP15 gene knockdown was checked using SDS-PAGE (15% (w/v) acrylamide resolving gel) and western blot analysis.

### WSSV gene expression analysis of *Pm*VRP15 knockdown *P. monodon* hemocytes infected with WSSV

WSSV-infected shrimp hemocyte after *Pm*VRP15 gene knockdown or GFP gene knockdown was prepared as above. The expression level of representative WSSV gene transcripts of the immediate-early, early and late viral infection phases was then evaluated in the hemocytes by qRT-PCR.

The qrt-PCR was then performed on the BioRad CFX96 Real-Time PCR system to evaluate the degree of the respective gene transcripts. Reactions were prepared in a total volume of 15 μL containing 7.5 μL SsoFast EvaGreen Supermix (Bio-Rad) and 1 μL cDNA template and 100 nM (for WSSV genes) or 400 nM (for EF-1α gene) forward and reverse primers.

For the expression level of the three WSSV transcripts (*ie*-1, *wsv477* and *vp28*), the qrt-PCR was performed using the specific primer pairs ie1-qrt-F/R, 477-qrt-F/R and VP28-qrt-F/R, respectively (Table 1), along with the EF-1α gene as a reference gene using the EF-1-F/R primers. The PCR conditions were 95°C for 5 min, followed by 40 cycles of 95°C for 30 s, 60°C (for all WSSV genes) or 58°C (for EF-1α) for 30 s and 72°C for 30 s. Three replicate qrt-PCR reactions were performed per sample. The 2^−ΔΔ*C*t^ method was used to calculate the relative expression ratio [25]. All samples were normalized relative to the reference EF-1α transcript levels in the same cDNA sample.

### Effect of *Pm*VRP15 gene silencing on cumulative mortality of WSSV-infected shrimp

To study the involvement of *Pm*VRP15 gene in WSSV infection in shrimp, the percentage of cumulative mortality of WSSV-infected *Pm*VRP15 knockdown shrimp was compared with WSSV infected GFP knockdown shrimp, control group. Ten *P. monodon* shrimps of approximately 3 g body weight per group were injected with *Pm*VRP15 dsRNA or GFP dsRNA as above. The dosage of WSSV used in this experiment causes 100% mortality of shrimps in 4 dpi. The shrimp mortality was observed every 3 h after WSSV infection. This experiment was done in triplicate. Moreover, after WSSV infection, shrimp hemocyte was collected at 24, 36, 48 and 60 hpi. Total RNA was extracted. After DNase treatment and cDNA synthesis, *Pm*VRP15 gene expression was investigated by RT-PCR in order to determine *Pm*VRP15 gene recovery.

### Data analysis

Data were analyzed using the SPSS statistics 17.0 software (Chicago, USA) and are presented as the mean ±1 standard deviation (SD). Statistical significance of differences between means was calculated by the paired-samples *t*-test, where significance was accepted at the *P*<0.05 level.

## Results

### The full-length cDNA of *Pm*VRP15 and sequence analysis

**Figure 1:**
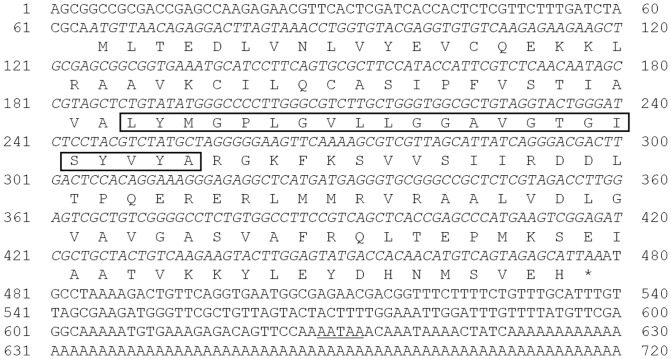
The cDNA nucleotide and deduced protein amino acid sequences of *Pm*VRP15 (GenBank accession code KF683338). The putative start codon (ATG) is in bold, the asterisk indicates the stop codon (TAA, in bold italics), the potential transmembrane domain is boxed and the proposed polyadenylation site is underlined.

A partial sequence of the *Pm*VRP15 cDNA was initially obtained from the SSH library of WSSV-challenged *P. monodon* hemocytes. The full-length cDNA of *Pm*VRP15 was then obtained using 5′ RACE (GenBank accession code KF683338), and was found to contain 722 base pairs with a deduced complete open reading frame encoding for a predicted 137 amino acids whose predicted molecular mass of 15.036 kDa (Fig. 1). The size of the deduced *Pm*VRP15 cDNA was confirmed by Northern blot analysis where the detected mRNA had a corresponding size of about 722 base pairs (data not shown). The BLAST homology search of the GenBank database using blastP program indicated that the putative predicted protein sequence of *Pm*VRP15 has the highest similarity to a hypothetical protein AGAP000432-PA (XP_310667.1) from mosquito *Anopheles gambiae* with a significant E value of 2e−11, 35% identity and 58% similarity. Lower significant similarity of *Pm*VRP15 with other five hypothetical proteins such as conserved hypothetical protein (XP_001849829.1) from *Culex quinquefasciatus*, GE16519 (XP_002099810.1) from *Drosophila yakuba*, GK10098 (XP_002071654.1) from *Drosophila willistoni*, hypothetical protein AaeL_AAEL014657 (XP_001649261.1) from *Aedes aegypti*, and PREDICTED protein C19orf12 homolog (XP_004536446.1) from *Ceratitis capitata*, was also found with the E value range from 10^−8^–10^−5^. Protein-structural analysis revealed a likely transmembrane helix of 23 amino acids (TMHMM Server v. 2.0, available on-line) [27] but with no predicted signaling domain (Simple Modular Architecture Research Tool (SMART), available on-line) [28].

### *Pm*VRP15 gene expression in *P. monodon* tissues

**Figure 2:**
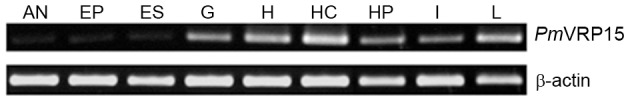
*Pm*VRP15 transcript expression analysis in various *P. monodon* tissues by RT-PCR. The tissues examined were antennal gland (AN), epipodite (EP), eye stalk (ES), gill (G), heart (H), hemocyte (HC), hepatopancreas (HP), intestine (I) and lymphoid (L). EF1-α was used as the internal reference and PCR control.

The tissue distribution of *Pm*VRP15 transcripts in normal shrimps was examined by RT-PCR, where *Pm*VRP15 transcripts were found in all tested tissues but was highly expressed in hemocytes followed by lymphoid tissue and then with moderate to low levels in the heart, gill, hepatopancreas and intestine, and low levels in the antennal gland, epipodite and eye stalk (Fig. 2).

### Up-regulation of *Pm*VRP15 in response to WSSV infection in *P. monodon* hemocytes

**Figure 3:**
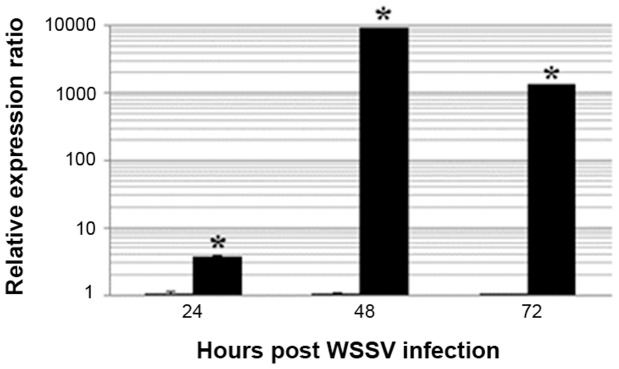
Up-regulation of *Pm*VRP15 transcripts in response to WSSV infection. Relative expression ratios, as determined by qRT-PCR, of *Pm*VRP15 transcript levels in the hemocytes of WSSV-infected *P. monodon* were compared to those of the control (non-infected) shrimps and standardized against β-actin as the internal reference, at 24, 48 and 72 hpi with WSSV. The data represent the mean ±1 SD relative expression of *Pm*VRP15 post-infection (solid bar, right) and the control (open bar, left), derived from three independent experiments. Means with an asterisk are significantly different (*P*<0.05, paired samples *t*-test). A relative expression ratio of <1, 1 and >1 mean that the gene expression level is down-regulated, the same or up-regulated, respectively, in the hemocytes of WSSV-infected shrimps compared to the uninfected control.

**Figure 4:**
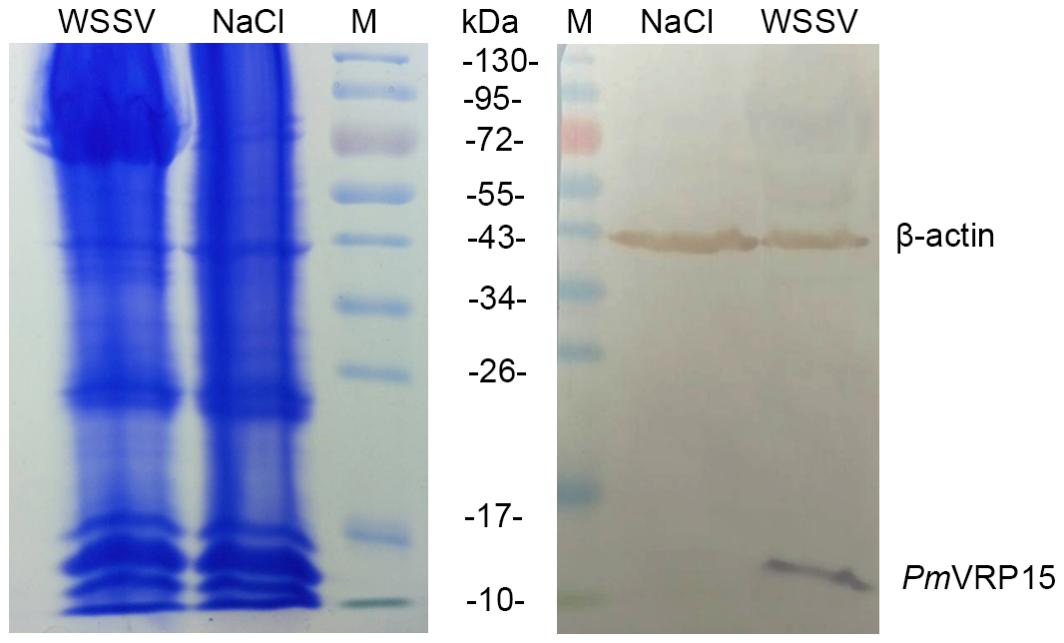
Western blot analysis of *Pm*VRP15 native protein in control (NaCl) and WSSV- injected (WSSV) *P. monodon* hemocytes. Hemocytes were collected at 48(HLS) was prepared and 70 μg of total HLS protein per track was subjected to duplicate SDS-PAGE resolution. Gels were then either stained with coomassie blue for total protein detection or subject to Western-blot analysis to detect *Pm*VRP15 and β-actin using specific antibodies. M is the protein size markers.

Our previous results from SSH and microarray analyses (unpublished data) of WSSV-challenged *P. monodon* hemocytes revealed that *Pm*VRP15 is one of the most highly up-regulated genes in the acute phase of WSSV-infected hemocytes. Herein, *Pm*VRP15 transcript levels in *P. monodon* hemocytes were evaluated by qRT-PCR. The results clearly confirmed that *Pm*VRP15 transcripts were highly up-regulated in the shrimp hemocytes after WSSV challenge, increasing by about 3.6-, 9410- and 1351-fold at 24, 48 and 72 hpi, respectively, compared to that in unchallenged shrimp hemocytes (Fig. 3). Furthermore, the *Pm*VRP15 protein level was up-regulated in WSSV-infected shrimp hemocytes, as determined by Western blot analysis using the polyclonal rabbit anti-r*Pm*VRP15 antibody (Fig. 4), where the detected 15 kDa protein band corresponded to the predicted size of *Pm*VRP15 protein. These results are consistent with a role for *Pm*VRP15 in response to WSSV infection.

### Localization of *Pm*VRP15 and VP28 in uninfected and WSSV-infected *P. monodon* hemocytes

**Figure 5:**
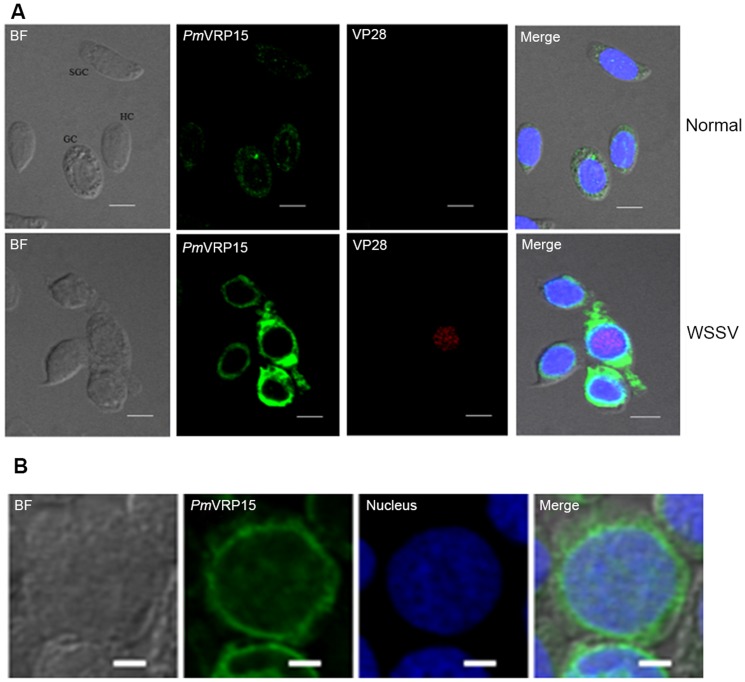
CFLM-derived images of the uninfected (control) and WSSV-infected hemocytes at 48 hpi with WSSV. Rabbit anti-r*Pm*VRP15 and mouse anti-VP28 primary antibodies were detected with corresponding Alexa488 and Alexa568 secondary antibodies revealing *Pm*VRP15 (green color) and VP28 (red color), respectively. Scale bars represent (A) 5 μm and (B) 2 μm. Nucleus was stained with TO-PRO-3 iodide and color was adjusted to blue. The bright field image showed hyaline cell (HC), semigranular cell (SGC) and granular cell (GC).

The location of *Pm*VRP15 and VP28 proteins in hemocytes and the potential cell type(s) that produce the protein was examined by CLSM using the antibodies specific to *Pm*VRP15 and the WSSV late protein VP28 coupled with different fluorescence-conjugated secondary antibodies. Since *Pm*VRP15 and VP28 were detected as green and red fluorescence, respectively, the accepted fraction of the emission spectra of TO-PRO-3, used to stain the nuclear DNA, was adjusted to show as blue. Three types of hemocytes (hyaline, semigranular and granular cells) were visible in the bright field image (Fig. 5A). In the uninfected (control) shrimp hemocytes, all three types of hemocytes were weakly positive for *Pm*VRP15, and the protein was localized in the cytoplasm near to the nuclear membrane (Fig. 5B). In the WSSV-infected shrimps, the *Pm*VRP15 protein expression level was hardly detected at 6 hpi but significantly up-regulated at 24 hpi (not shown) and 48 hpi (Fig. 5) and in the moribund shrimps (not shown). Interestingly, *Pm*VRP15 and VP28 protein expression were found in the same hemocytes at the late infection phase (48 hpi) and the moribund stage of viral infection (Fig. 5 and not shown, respectively). Thus, the expression of *Pm*VRP15 in *P. monodon* hemocytes appears to be linked to a response to the acute phase of WSSV infection.

### Effect of *Pm*VRP15 gene knockdown on viral propagation in *P. monodon* hemocytes

**Figure 6:**
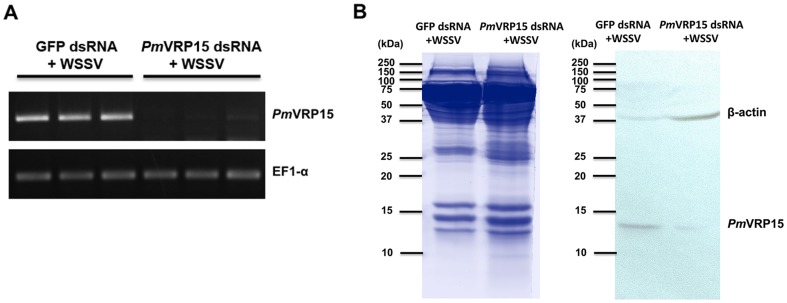
The *Pm*VRP15 gene silencing in *P. monodon* hemocytes. (A) Transcriptional level of *Pm*VRP15 transcripts after 24 h post-WSSV infection and -*Pm*VRP15 gene knockdown in the *P. monodon* hemocytes was determined by RT-PCR using gene specific primers. The control was shrimp that was injected with GFP dsRNA. Three individuals were used for each group and each experiment was performed in triplicate. (B) Protein expression level of *Pm*VRP15 was detected in both groups to confirm the success of *Pm*VRP15 knockdown. Hemocytes were collected at 24 h after *Pm*VRP15 gene knockdown in WSSV-infected shrimp, 70 μg of total HLS protein was analyzed by SDS-PAGE and western blot analysis using antibody specific to *Pm*VRP15 and β-actin protein, an internal control.

Since *Pm*VRP15 transcripts and protein were found to be highly up-regulated in the hemocytes of WSSV-infected shrimps, then the potential importance of *Pm*VRP15 in the shrimp's response to WSSV infection was evaluated using RNAi-mediated gene knockdown by injection of *Pm*VRP15 dsRNA. Injection of dsRNA *Pm*VRP15 specifically suppressed the *Pm*VRP15 transcription levels in shrimp hemocytes at 24 hpi whereas the injection with GFP dsRNA had no effect on *Pm*VRP15 mRNA expression levels (Fig. 6A). The suppression of *Pm*VRP15 expression at the translational level was also confirmed. Twenty-four hour after knocking-down *Pm*VRP15 gene in WSSV-infected shrimp, *Pm*VRP15 protein expression level in the shrimp hemocyte lysate was compared with that of the control WSSV-infected shrimp with GFP dsRNA injection. The result showed that *Pm*VRP15 protein expression level in WSSV-challenge shrimp was significantly decreased after *Pm*VRP15 gene silencing (Fig. 6B).

**Figure 7:**
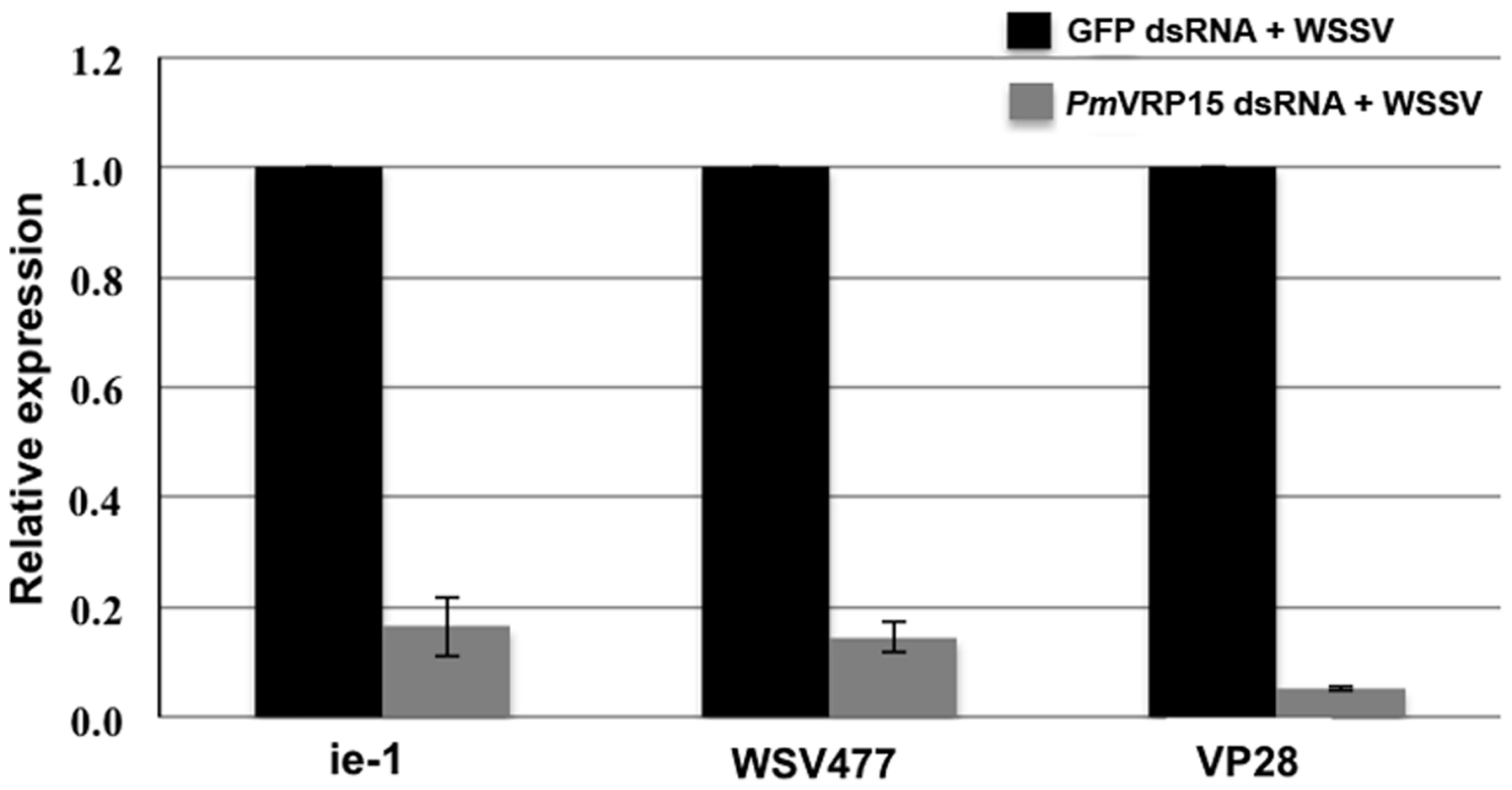
The effect of *Pm*VRP15 gene silencing on WSSV propagation in *P. monodon* hemocytes. Transcript expression level of the WSSV genes: *ie-1*, *wsv477* and *vp28*, in *Pm*VRP15 gene-silenced *P. monodon* hemocytes were determined by qRT-PCR. Data are shown as the mean ±1 SD of three replicates and as the fold change of *ie-1*, *wsv477* and *vp28* after normalization to the EF-1α transcript levels (grey bar). The control group (GFP-dsRNA injected) are shown in the black bars.

The transcript expression level of representative WSSV genes for the three stages of WSSV infection; namely *ie*-1 (very early stage), *wsv477* (early stage) and *vp28* (late stage), was determined after *Pm*VRP15 knockdown in WSSV-infected shrimp by qRT-PCR. The transcript expression level of all three viral genes tested was considerably decreased in the *Pm*VRP15 knockdown shrimps (by 83.5%, 85.5% and 94.8% for *ie-*1, *wsv477* and *vp28*, respectively) compared to the control shrimps (Fig. 7). The decrease in WSSV transcript levels suggested that *Pm*VRP15 might participate in the WSSV propagation process.

### Cumulative mortality of *P. monodon* shrimp after *Pm*VRP15 gene knockdown

**Figure 8:**
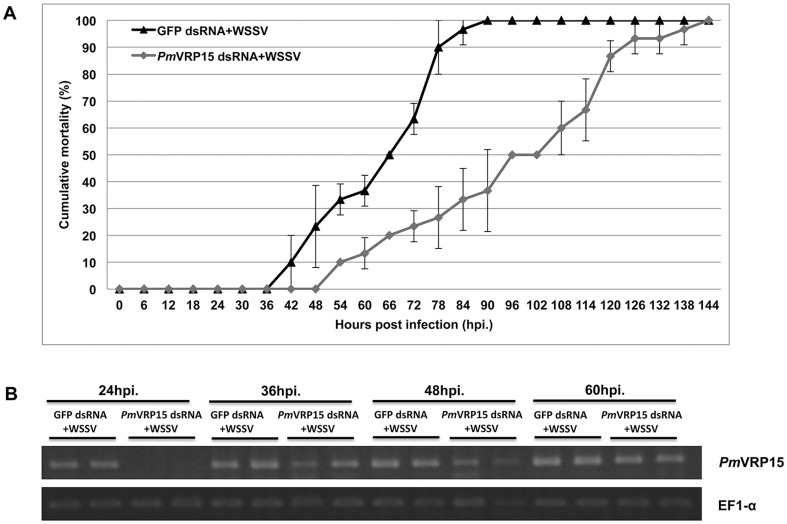
The involvement of knockdown *Pm*VRP15 gene in WSSV infection in shrimp. (A) Cumulative mortality of WSSV-infected *Pm*VRP15 gene knockdown shrimp (black line) was compared with that of the control, WSSV-infected GFP gene knockdown shrimp (Grey line). Data are shown as the mean ±1 S.D. and are derived from three independent repeats. (B) After knockdown *Pm*VRP15 gene in WSSV-infected shrimp, *Pm*VRP15 gene recovery was observed after WSSV infection at 24, 36, 48 and 60 hpi.

As stated above, after *Pm*VRP15 gene knockdown in WSSV-infected shrimp, the expression level of representative WSSV genes was significantly decreased suggesting the involvement of *Pm*VRP15 in the WSSV propagation. *Pm*VRP15 gene was silenced in WSSV-infected *P. monodon* and the mortality of shrimp was observed in parallel to those silenced with GFP dsRNA. The cumulative mortality result showed that, after 66–102 hours post-WSSV infection, mortality rate of *Pm*VRP15 knockdown group was 50% lower than that of control group (The shrimp mortality reached 100% at 90 hpi) (Fig. 8A). However, after 102 hpi, the cumulative mortality of *Pm*VRP15 knockdown shrimp was gradually increased and reached 100% at 144 hpi (6 dpi). Due to the fact that *Pm*VRP15 gene is highly up-regulated after WSSV infection, here, the *Pm*VRP15 gene recovery after *Pm*VRP15 dsRNA and WSSV injection was determined. Figure 8B showed that *Pm*VRP15 gene was recovered for about 50% at 36 hpi and to the same level as in the control at 60 hpi. According to the results, we confirmed that the absence of *Pm*VRP15 gene in shrimp affected the mortality of WSSV-infected shrimp.

## Discussion

To study the mechanism of WSSV infection and propagation, an understanding of the immune response of shrimps is an important key. *Pm*VRP15 transcripts were found in all tissues examined of uninfected *P. monodon* shrimps, but were mainly expressed in the hemocytes. Hemocytes are the major immune cells of shrimps and play an essential role in both the cellular and humoral immune responses. Three different types of hemocytes (granular, semigranular and hyaline cells) have been classified in shrimp hemolymph [29]–[30]. In crustaceans, specific (but partially overlapping) functions have been attributed to the different hemocyte types, such as phagocytosis in hyaline cells, encapsulation, phagocytosis, ProPO system and cytotoxicity in semigranular cells, and the ProPO system and cytotoxicity in granular cells [31]. In contrast, the *Pm*VRP15 protein was located in all three types of hemocytes, suggesting that *Pm*VRP15 may have a more constitutive or broad immune based function. Upon WSSV infection, the expression of *Pm*VRP15 transcripts and protein were both up-regulated in *P. monodon* hemocytes, exclusively within WSSV-infected ones.

From the SSH analysis (our unpublished data), *Pm*VRP15 transcripts appeared to be highly expressed in the acute phase of WSSV-infected *P. monodon* hemocyte; however, the function of its gene product have not been characterized. Interestingly, several hemocyte proteins were found to be significantly altered in their expression levels in the different stages of virus infection, including both well-characterized proteins and those of currently unknown function [21]. Several cognate immunity proteins involved in viral defense responses have been found to be up-regulated in the early phase of viral infection, such as the antimicrobial peptides ALF*Pm*3, Peneidin5 and hemocyanin [21], [32]–[34]. During the acute phase of WSSV infection, the host immune responses and mechanism(s) used are not yet fully understood, but several host proteins have previously been identified that show altered expression levels, including the scavenger receptor [35] and transglutaminase [36] amongst others. Recently, the unknown function *Pm*HHAP, which is highly up-regulated in viral infected shrimps, was identified and characterized as a novel responsive protein that plays an important role in hemocyte homeostasis [23].

Nuclear membrane proteins have been reported in many vertebrates to act as a path of infection for viruses, such as influenza virus [37] and herpes virus [38]–[39]. However, such protein functions remain unknown in invertebrates. Herein, we found that the expression of *Pm*VRP15 was mainly located at the nuclear membrane of *P. monodon* hemocytes. Moreover, hemocytes that were infected with WSSV also expressed *Pm*VRP15 at high levels. It would be interesting to study how a severe WSSV infection can stimulate *Pm*VRP15 expression. In addition, the data presented here may represent the first report linking a correlative relationship between a potential *P. monodon* nuclear membrane protein (*Pm*VRP15) and WSSV infection. However, an actual direct causative role, and the mechanism of such, remains to yet be established.

In the acute viral infection phase, the host cells not only express defensive molecules that play a role in protecting the host cell against the virus, but the virus uses the host machinery to express viral proteins for propagation, including the immediate early, early and late genes [40]. At this stage the host cell loses the ability to regulate gene expression and is seconded to perform virus multiplication. Although cell death by apoptosis is one last line of host defense, whereby the infected cell is self-signaled for destruction to prevent viral replication and so to protect against viral spread to other cells, some viral proteins can inhibit the apoptosis system, including in WSSV the anti-apoptosis protein-1, AAP-1 [41] and WSSV222 [42]. The high expression level of *Pm*VRP15 found here in WSSV-infected *P. monodon* hemocytes is in agreement with (but not conclusive for) that *Pm*VRP15 is up-regulated to mediate viral propagation in the acute phase of infection, since *Pm*VRP15 gene knockdown resulted in a significant decrease in viral gene expression, as observed for *ie*-1 (an immediate early gene), *wsv477* (an early gene) and *vp28* (a late gene) transcripts and in the delay of shrimp death upon WSSV infection. Additionally, *Pm*VRP15 protein was found to be localized near the nuclear membrane in the cytoplasm of WSSV-infected hemocytes which coupled with the predicted presence of transmembrane domain, suggests it may function at least in part as a nuclear membrane (or proximally related membrane) protein. If so, this is in accord with the notion that the host machinery was used to transport the viral components in the host cell, as found in the transmembrane protein *Pm*Rab7 [43]. The interaction of viral and host proteins is a potentially important key to answer the function of *Pm*VRP15 in WSSV-infected cells, and could initially be addressed by, for example, using co-immunoprecipitation or the yeast two-hybrid screening assays. Nevertheless, the mechanism of control of expression (transcriptional control) of the *Pm*VRP15 gene would also be interesting to elucidate, including characterization of the promoter. These aspects are now under investigation in an attempt to reveal the mechanism and regulation of *Pm*VRP15 in WSSV propagation in *P. monodon* hemocytes.

## Conclusion

The cDNA of a novel viral responsive gene from the black tiger shrimp (*P. monodon*), *Pm*VRP15, was cloned and sequenced to acquire the full-length cDNA coding sequence. Expression analysis showed *Pm*VRP15 transcripts were mainly found in hemocytes and along with the *Pm*VRP15 protein were highly up-regulated in WSSV-infected hemocytes. *Pm*VRP15 protein was localized at or near the nuclear membrane of uninfected and WSSV-infected shrimp hemocytes. After RNAi-mediated *Pm*VRP15 suppression, WSSV propagation and shrimp mortality were markedly decreased. The function of *Pm*VRP15 is unknown but it possibly plays a role in WSSV propagation in shrimp hemocyte.
