# Correction: *Pm*VRP15, a Novel Viral Responsive Protein from the Black Tiger Shrimp, *Penaeus monodon*, Promoted White Spot Syndrome Virus Replication

**DOI:** 10.1371/journal.pone.0106274

**Published:** 2014-08-15

**Authors:** The PLOS ONE Staff

The second and third authors, Adisak Prapavorarat and Phattarunda Jaree, should be noted as contributing equally to this work.

## References

[1] Vatanavicharn T, Prapavorarat A, Jaree P, Somboonwiwat K, Tassanakajon A (2014) *Pm*VRP15, a Novel Viral Responsive Protein from the Black Tiger Shrimp, *Penaeus monodon*, Promoted White Spot Syndrome Virus Replication. PLoS ONE 9(3): e91930 doi:10.1371/journal.pone.0091930 24637711 10.1371/journal.pone.0091930 PMC3956821

